# Does capitation affect patient satisfaction and prevalence of out-of-pocket payments in the insured? A propensity score analysis of Ghana’s demographic and health survey data

**DOI:** 10.1186/s12913-019-4581-4

**Published:** 2019-10-22

**Authors:** Shafiq Siita, Sharon E. Cox, Kara Hanson

**Affiliations:** 1 National Health Insurance Authority, Upper East Regional Office, Bolgatanga, Ghana; 2 School of Tropical Medicine and Global Health, Nagasaki University, Nagasaki, Japan; 3 London School of Hygiene and Tropical Medicine (LSHTM), London, UK

**Keywords:** Capitation, Out-of-pocket, Quality perceptions, Propensity score, Health insurance, Ghana

## Abstract

**Background:**

Ghana’s National Health Insurance Scheme (NHIS) piloted capitation payment for primary care services in the Ashanti region from 2012 to 2017. Capitation was piloted as a means of cost containment but also to induce managed competition among health providers to improve the responsiveness of healthcare delivery. This study examined the effects of exposure to capitation on perceived health service quality and prevalence of out-of-pocket payments in NHIS insured clients.

**Methods:**

Respondents of the 2014 Ghana Demographic and Health Survey (G-DHS) who reported having a valid NHIS card as their only form of health insurance coverage and made a health facility visit within the 6 months prior to the survey were used to assess the exposure effects of capitation on four outcomes: overall patient satisfaction, perceived friendliness of health staff, perceived adequacy of consultation time, and prevalence of out-of-pocket payments. We applied propensity score matching to balance distributions of covariates and to compare outcomes between exposed NHIS insured clients and their unexposed counterparts.

**Results:**

NHIS insured clients exposed to capitation had 10 percentage points higher probability of encountering out-of-pocket payments than their unexposed counterparts (*p* = 0.009; 95% CI: 2.5–17.8%). There was no evidence of a difference between the two exposure groups for ratings of the three quality perceptions outcomes examined: overall patient satisfaction, difference 0.63 units (*p* = 0.46); perceived friendliness of health staff, difference 1.1% (*p* = 0.50); and perceived adequacy of consultation times, difference 0.1% (*p* = 0.96).

**Conclusion:**

In the Ghanaian context, our results suggest capitation was associated with a greater probability of out-of-pocket payments and no difference in perceived service quality. Future research should examine clinical quality of healthcare and how much out-of-pocket payment occurred under capitation.

## Background

In the face of escalating healthcare costs and limited financial resources, the role of strategic purchasing to improve health system performance and facilitate progress towards universal health coverage has become increasingly critical. One of the key components of strategic purchasing is how health providers are paid for contracted services [1]. This determines their exposure to financial risk, the incentives they face, and consequently, how they respond in terms of health service delivery [2]. Health purchasing agencies therefore often use provider payment methods to influence provider behaviours in ways that promote efficiency, quality and responsiveness [3]. Ghana’s National Health Insurance Scheme (NHIS), in its efforts to move away from passive to more strategic purchasing, has been experimenting with alternative payment methods to achieve cost-efficient healthcare delivery to its beneficiaries without compromising healthcare quality. The latest payment reform, following fee-for-service and diagnostic related groupings (DRG), was the pilot of capitation in the Ashanti region from 2012 to 2017.

The capitation pilot was for the payment of non-specialist primary care outpatient services. All NHIS contracted health facilities within the Ashanti region participated in the payment reform while facilities in the other nine regions remained under the existing Ghana Diagnostic Related Grouping (G-DRG) payment method for both outpatient and inpatient services. Per capita rates were prospectively paid to health facilities to provide the capitated basket of services for their enrolled clients for a period of 1 month. The capitated rate was not risk adjusted but was adjusted to reflect health facilities’ ownership. This was to account for supply-side subsidies that public and quasi-government facilities receive from government, mainly in the form of salaries and infrastructure. Medicines and inpatient services were excluded from the basket of capitated services and continued to be paid for using itemized fee-for-service and G-DRG [4]. Insured clients in the capitated region freely and actively selected their health facility, known as their Preferred Primary-care Provider (PPP) [5], and had the option to change their PPP at six-month intervals [6].

Except for emergency cases, insured clients in the capitated region were required to first visit their PPP for medical services and if necessary be referred by their PPP to other appropriate NHIS contracted health facilities [6]. Consequently, insured clients who visited other health facilities for non-emergency outpatient healthcare without proper referral from their PPP were liable for the cost of care [6]. As capitation payment is based on the number of insured clients enrolled with a service provider, health providers’ outpatient services revenue in the capitated region was largely a product of the per capita rate and the number of insured enrolled members, plus the cost of medicines dispensed to insured clients who sought care.

Theoretically, capitation serves as a cost control tool by shifting some financial risk to health service providers and thus encouraging them to deliver care in a more cost-efficient manner [7]. However, the financial risk imposed by capitation may present incentives to service providers to undersupply healthcare [7] or provide sub-standard care to reduce cost. Furthermore, in settings where there may be inadequate controls and weak monitoring mechanisms, capitation may lead to other unintended consequences such as providers demanding informal payments from insured clients in an attempt to offset part of the risk.

While cost containment was the main driver for its implementation [4], the NHIS implemented capitation to also “introduce managed competition in providers and choices for patients as a way of increasing the responsiveness of [Ghana’s] health system” [8]. Evidence from both industrialized and resource limited settings suggests some cost containment effects of capitation [9–12]. However, there is limited empirical evidence on the effects of capitation on health service quality and out-of-pocket payments among the insured. In Thailand, Tangcharoensathien and colleagues [13] found that insured clients whose outpatient services were paid for by capitation were less likely to be satisfied in most of the perceived quality outcomes the authors studied. Using regression analysis, Andoh-Adjei et al. [14] found no significant difference between NHIS insured clients in Ashanti region (where capitation was piloted) and those in two other regions in their likelihood of rating their perception of quality of care as “good”. This study builds on the previous studies by using a sub-sample from a nationally representative survey and applying a quasi-experimental methodology (propensity score matching) to examine the effects of capitation on perceived health service quality and prevalence of out-of-pocket health payments using Ghana’s capitation pilot as a case study.

## Methods

### Data sources

The study used data from the 2014 Ghana Demographic and Health Survey (G-DHS), the sixth and most recent survey. Details of the survey design and sampling strategy are described elsewhere [15]. Briefly, the survey stratified Ghana into urban and rural strata and sampled 427 enumeration areas (geographic demarcations of sets of households) from these strata at the first stage while 12,831 households were systematically sampled from the enumeration areas at the second stage [15]. At the household level, all women aged 15–49 in all selected households were eligible for interview while in half of the selected households, all men aged 15–59 were eligible. A total of 13,784 interviews in 9396 women and 4388 men were completed across the country [15]. The 2014 survey collected a broad range of data on health and health insurance coverage including whether the respondents had a valid NHIS card at the time of the survey and whether respondents made a health facility visit within the preceding 6 months. For those who made a health facility visit, the survey collected information on the kind of health services received, perceived quality of services, and method of payment.

The current study included all men and women who reported having a valid NHIS card as their only form of health insurance coverage and made a health facility visit within the 6 months prior to the survey for outpatient services covered by NHIS.

### Dependent and independent variables

Four main outcomes were evaluated: whether respondents experienced an out-of-pocket payment during the reported health facility visit and three perceived quality outcomes - patients’ overall satisfaction level, perceived adequacy of consultation time, and perceived friendliness of service providers. Respondents’ overall level of satisfaction was constructed as the sum of a 15-item scale (Additional file 1: Table S1): measuring different dimensions of health service quality on a five-point Likert scale. Cronbach’s alpha for internal consistency of the scale was 0.90, which is considered high [16]. The robustness of the results was evaluated using principal component analysis (PCA) to compute satisfaction index.

For perception of adequacy of consultation time, the survey asked respondents; “*In your opinion, did the health provider spend enough time with you?*”, to which they could answer “*Yes*” or “*No*”. Similarly, for perceived friendliness of health staff, respondents were asked “*Was the health provider friendly to you?*”. For the occurrence of out-of-pocket payments, respondents were asked “*How did you pay for the service during this most recent visit?*” and they could answer “*Cash*”, “*National Health Insurance*”, “*Other insurance*”, “*Combination of any of the above*” or “*Other*”. Since the study’s sub-sample comprised only respondents who reported having NHIS as their only health insurance coverage, responses of “*Other*” (10 observations) were recoded to missing and responses of “*Cash*” or “*Combination of any of the above*” were coded as out-of-pocket payments.

The main independent variable of interest was exposure to the capitation pilot in the Ashanti region. The exposed group was defined as insured respondents who reported residing in the Ashanti region at the time of the survey while those who reported residing outside the Ashanti region, and therefore whose outpatient services were paid for under G-DRG, were deemed unexposed.

### Analytical approach

We employed propensity score matching [17, 18] to balance relevant covariate distributions between the two exposure groups and to estimate the exposure effects of capitation on the outcomes for those who were exposed, defined as E [Y (1) – Y (0)|T = 1]. Where Y (1) is the observed outcome, Y (0) the counterfactual outcome and T the treatment status. Two critical assumptions are required for correct estimation of the exposure effect of capitation using this approach. First, exposure to capitation and the potential outcomes are assumed to be independent conditional on the observed covariates and second, every respondent is assumed to have a positive probability of being in either exposure group - positivity or overlap assumption [17]. If these two assumptions hold, matching on the propensity score theoretically removes bias from observed covariates [17, 19, 20] and thus, allows for an unbiased estimate of treatment effect.

There is limited methodological guidance on how to properly accommodate survey weights from complex survey designs in the context of propensity score matching [21]. Particularly, to the best of our knowledge, when population inferences are of interest, it is not clear how to properly account for both the fact that the propensity scores are estimated and the complex survey weights in estimating standard errors. Thus, we limited our study to sample inferences and estimated sample average treatment effect on the treated (SATET), which does not need adjustments for complex survey weights [22].

We followed steps recommended by Caliendo and Kopeinig [23] for the application of propensity score matching to estimate treatment effect. First, logit models were used to estimate the exposure probabilities and respondents were matched based on similarities of their exposure probabilities. Second, post-matching balance diagnostics were performed to assess the balance of covariates distributions between the exposed and unexposed. Third, the resultant matched sample was used to estimate the SATET. Finally, sensitivity analysis was conducted to explore the robustness of the findings against the assumption that no unmeasured confounders influenced exposure allocation.

### Estimation of propensity score and matching

Identification of covariates for the exposure probability model was guided by review of the existing literature [24] and only variables deemed to be potential confounders [25], but were not themselves likely to have been affected by capitation exposure [23, 26] were considered. From the literature, type of facility visited, place of residence, level of education, wealth, health status, and ethnicity were found to be correlates of health service quality perceptions [13, 27–30]. Self-reported clinician-diagnosed hypertension and household wealth measurements were used as proxies for respondent’s health status and wealth measurements respectively. The G-DHS used household assets data and dwelling characteristics to construct the wealth index and quintiles using principal component analysis [15]. Other key socio-demographic characteristics including age, gender, and religion were also considered in building the propensity score model but only age was included in the final model. It was initially planned to compute separate propensity score models for quality perception and out-of-pocket payment outcomes. However, the literature review revealed that most variables associated with quality perception were also associated with out-of-pocket payments. Thus, a single unweighted propensity score model was estimated for all outcomes.

A nearest-neighbour matching, with replacement, procedure was then implemented using the estimated exposure probabilities. We implemented a 1:1 matching structure but allowed exposed respondents to be matched with all closest unexposed respondents with identical propensity scores (ties). To improve the quality of matching, we imposed the condition that the maximum absolute difference between the exposure probabilities of matched respondents was not more than 0.09. This matching algorithm produced the best balance of covariates between the exposure groups with no loss of observations to common support.

### Post-matching balance diagnostics

Similarities of covariate distributions between exposure groups in the matched sample were assessed using absolute standardized mean differences (bias) and variance ratios [31, 32]. After matching, the standardized differences in covariate means are expected to be zero (or close to zero) and the variance ratios are expected to be 1 (or close to 1) if there is adequate balance of covariates. While there appears to be no clear standard for optimum balance of covariates [23], a standardized mean difference greater than 0.1 is commonly cited as indicative of substantial imbalance between groups [33, 34]. Rubin [34] further argues that variance ratios of covariates outside the range of 0.8 and 1.25 reflect large differences between groups such that regression adjustments for such covariates may not be reliable. Thus, a covariate was considered balanced if its standardised mean difference was less than absolute 0.1 and its variance ratio was between 0.8 and 1.25.

### Estimation of capitation effects on the outcomes

The effects of capitation were estimated by comparing the outcomes between the exposed and unexposed in the matched sample. We used Stata’s *teffects psmatch* command for the effect estimations. This estimator takes into account that the propensity scores are estimated in computing standard errors [35]. Additionally, we computed robust standard errors to account for any clustering of respondents due to the survey design. All effect estimates were evaluated at the 5% significance level.

### Sensitivity analysis

We conducted sensitivity analysis on the effect estimates using Rosenbaum-bounding approach [23, 36]. In summary, if there were no uneven influence of unmeasured confounders, then a matched pair with the same covariates would have equal odds of exposure allocation and their odds ratio (Γ) would be equal to one [23]. By increasing the value of Γ to reflect an inequality in the odds of exposure assignment (Γ > 1), one can examine the degree of influence that an unmeasured confounder must have on the selection process to affect a study’s inferences [23, 37]. For the outcomes with evidence of a difference between the exposure groups, sensitivity analyses were conducted by examining different values of Γ > 1, at increments of 0.1, to determine up to which value of Γ a significance level of 0.05 was maintained. Inferences are considered sensitive to hidden bias if values of Γ closer to 1 alter the results, and are considered relatively robust if larger values of Γ are required to obtain different results [38]. We used Stata’s *mhbounds* [36] for the analysis.

Given that effect estimates can be affected by the kind of matching algorithm implemented, we also used a different matching estimator (Stata’s *teffects nnmatch*) to check the sensitivity of the effect estimates. Stata’s *teffects nnmatch* estimator is nonparametric and uses the Mahalanobis distance [19] to measure similarities between subjects. The same variables used for estimating the propensity score were used for the Mahalanobis distance matching. We did not impose a maximum distance at which subjects were a potential match. Any maximum distance below six (6) resulted in some loss of exposed respondents to common support.

We used Stata statistical software (version 14.2) to conduct all analyses and construct all figures.

## Results

### Background characteristics

Of the 13,784 respondents, 2256 met the selection criteria out of which 16 respondents were excluded due to missing data. Of the remaining 2240 respondents, 176 (8%) were exposed to capitation while 2064 (92%) were unexposed. Table 1 presents the distributions and unadjusted comparisons of background characteristics between the exposure groups in the unmatched sample. The sample was predominately female with 84% (1879) of respondents in the overall sample being women and no difference between the exposed and unexposed groups. Except for gender and most age groups, systematic differences between the exposure groups were observed in all the covariates. Fifteen (15) of the covariates recorded absolute standardized mean differences of more than 0.1 (10%) while 12 of them recorded variance ratios of less than 0.8 or more than 1.25.

**Table 1:** Comparison of background covariates between exposure groups in the unmatched sample

Mean						
Variable	Overall (*N* = 2240)	Exposed (*N* = 176)	Unexposed (*N* = 2064)	*P*-value^a^	Standardized Difference	Variance Ratio
(1)	(2)	(3)	(4)	(5)	(6)	(7)
Gender						
Female	0.839	0.824	0.840	Ref.	–	–
Male	0.161	0.176	0.160	0.573	0.043	1.086
Age Group						
15–19	0.113	0.125	0.112	0.613	0.039	1.102
20–24	0.163	0.131	0.166	0.227	−0.099	0.826
25–29	0.201	0.188	0.202	0.644	−0.037	0.950
30–34	0.165	0.188	0.163	0.396	0.065	1.124
35–39	0.143	0.119	0.145	0.344	−0.077	0.850
40–44	0.103	0.142	0.099	0.073	0.131	1.369
Over 44 yrs.	0.112	0.108	0.112	0.857	−0.014	0.970
Level of Education						
None	0.227	0.085	0.239	< 0.001	− 0.426	0.431
Primary Education	0.157	0.091	0.163	0.012	−0.217	0.610
Secondary/Higher	0.616	0.824	0.598	< 0.001	0.513	0.607
Wealth Quintile						
Poorest	0.245	0.051	0.262	< 0.001	−0.605	0.252
Poorer	0.170	0.097	0.176	0.007	−0.234	0.604
Middle	0.203	0.142	0.208	0.036	−0.175	0.743
Richer	0.200	0.318	0.190	< 0.001	0.296	1.415
Richest	0.181	0.392	0.163	< 0.001	0.527	1.754
Ethnicity						
Akan	0.365	0.653	0.341	< 0.001	0.658	1.014
Ewe	0.136	0.017	0.146	< 0.001	−0.484	0.135
Mole-Dabgani	0.282	0.153	0.293	< 0.001	−0.339	0.631
Other	0.217	0.176	0.221	0.167	−0.112	0.847
Type of Residence						
Rural	0.479	0.324	0.492	Ref.	–	–
Urban	0.521	0.676	0.508	< 0.001	0.347	0.881
Ever been told by a clinician as having hypertension						
No	0.889	0.858	0.892	Ref.	–	–
Yes	0.111	0.142	0.108	0.168	0.103	1.271
Type of facility visited						
Public/Government	0.831	0.688	0.843	Ref.	–	–
Private	0.169	0.313	0.157	< 0.001	0.373	1.632
Outcome Variables						
Out-of-pocket payments	0.146	0.301	0.132	< 0.001	–	–
Patient Satisfaction	59.439	60.756	59.327	0.031	–	–
Perceived friendliness of staff	0.948	0.966	0.946	0.260	–	–
Perceived adequacy of consultation time	0.926	0.915	0.927	0.541	–	–

Note: The analyses are not weighted; ^a^Two-sample t-test comparisons of means between exposed and unexposed

### Propensity score estimation and balance diagnostics

Results for the exposure probability model are reported in (Additional file 1: Table S2). Wealth quintile and visiting a private facility for healthcare were positively associated with exposure after adjusting for other covariates. Conversely, holding other covariates constant, respondents of other ethnic groups and those residing in urban areas were less likely to be exposed compared to their reference groups of being an Akan and residing in rural areas.

**Fig. 1:**
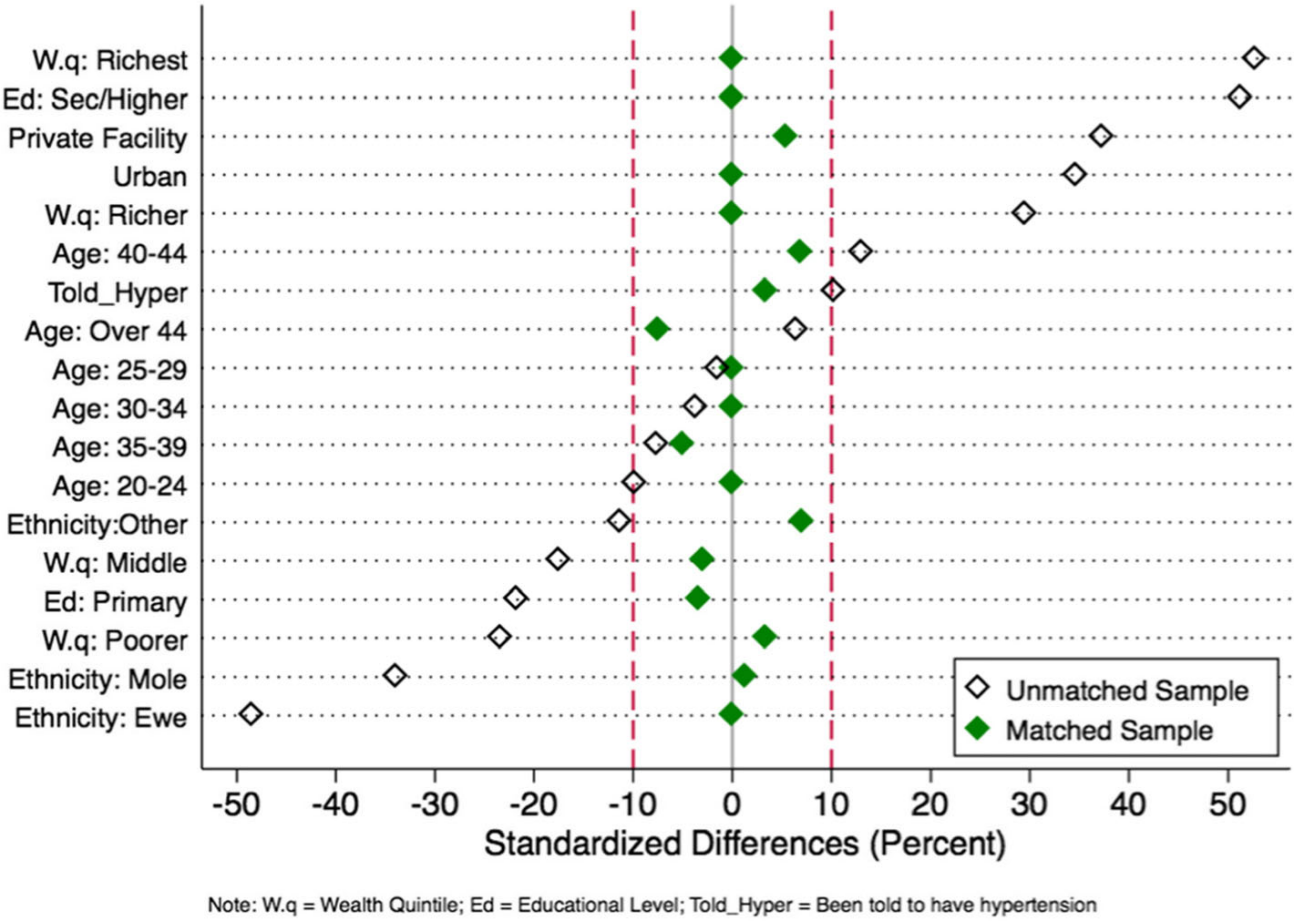
Covariates balance before and after matching

**Fig. 2:**
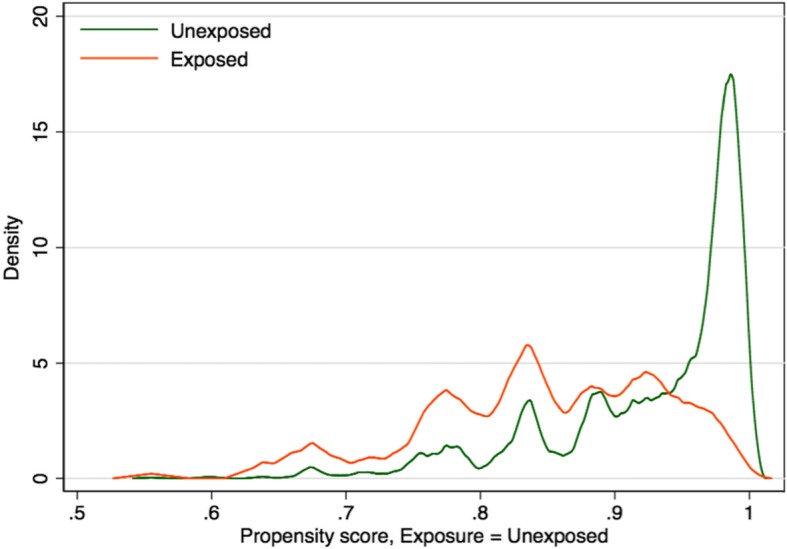
Exposure probability densities of exposure groups

All the exposed respondents found matches of unexposed counterparts. Matching eliminated or substantially reduced, in absolute terms, the systematic differences in covariates observed in the unmatched sample. After matching, all the covariates had absolute standardized mean differences less than 10% (Fig. 1) and their variance ratios were all between 0.8 and 1.25 (Additional file 1: Table S3). An assessment of the overlap assumption is presented in Fig. 2. Though the unexposed density plot showed a spike of probability mass near 1, the two density plots overlap over a wide range on the x-axis suggesting no evidence of a violation of this assumption.

### Effects estimates of capitation on outcomes

Estimates of the effects of capitation on the four outcomes are presented in Table 2. For prevalence of out-of-pocket payments, the unadjusted estimates in the unmatched sample suggested that exposed NHIS insured patients were on average, about 17% more likely to experience out-of-pocket health payments compared with their unexposed counterparts (*p* < 0.001; 95% Confidence interval [CI]: 11.5–22.3%). After balancing the distribution of covariates between the two groups, the magnitude decreased to 10 percentage points but remained significant (*p* = 0.009; 95% CI: 2.5–17.8%).

**Table 2:** Exposure effects of capitation on outcomes

Outcome	Before Matching			After Matching					
	Crude^a^ Mean Diff.	t-stat	*P*-value	SATET^b^	S.E.^c^	z-stat	*P*-value	95% Confidence Interval	
Prevalence of out-of-pocket	0.169	6.15	< 0.001	0.101	0.039	2.61	0.009	0.025	0.178
*Perceived Quality Outcomes*									
Patients’ Satisfaction	1.429	2.16	0.031	0.625	0.854	0.73	0.464	−1.048	2.298
Perceived friendliness of health staff	0.020	1.13	0.260	0.011	0.017	0.68	0.499	−0.021	0.044
Perceived adequacy of consultation time	−0.013	− 0.61	0.541	0.001	0.025	0.05	0.956	−0.048	0.051

^a^Unadjusted mean differences; ^b^Sample average treatment effect on the treated; ^c^Robust standard errors

There was no evidence of a difference between the two exposure groups in any of the perceived quality outcomes after matching. Before matching, the exposed group had on average, a statistically significant 1.43 units higher overall satisfaction experience compared to the unexposed (*p* = 0.031). This estimate however, diminished after matching to 0.63 units with a confidence interval that includes zero (*p* = 0.464). The estimated differences for both perceived friendliness of health staff and perceived adequacy of consultation time between the two exposure groups were neither statistically significant in the unmatched sample nor in the matched sample.

### Sensitivity analysis

Table 3 presents the Rosenbaum-bounds sensitivity analysis for the prevalence of out-of-pocket estimate. Under the assumption of no uneven influences of unmeasured confounders, the odds ratio (Γ) is 1 and the effect estimate is significant. However, if there were unmeasured confounders such that insured clients exposed to capitation were more likely to experience out-of-pocket payment even without capitation and given that they have the same observed covariates as the comparison group, then the current estimate would have over-estimated the exposure effects of capitation. Under the assumption of over estimation, an unobserved variable would have to influence the odds ratio of exposure allocation to differ between the two exposure groups by a factor of more than 1.4 to make the estimate non-significant at the 5% level and by a factor of more than 1.5 to render it non-significant at the 10% level (Table 3). However, assuming an under estimation, the estimate remained significant at all considered levels of Γ suggesting that it was unlikely to be an underestimate.

**Table 3:** Rosenbaum-bounds sensitivity analysis for estimate of prevalence of out-of-pocket payments

Gamma (Γ)	Mantel-Haenszel Test Statistic		Significance level	
	Assumption: Over-estimation	Assumption: Under-estimation	Assumption: Over-estimation	Assumption: Under-estimation
1.0	3.467	3.467	< 0.001	< 0.001
1.1	2.974	3.970	0.001	< 0.001
1.2	2.525	4.432	0.006	< 0.001
1.3	2.115	4.862	0.017	< 0.001
1.4	1.737	5.265	0.041	< 0.001
1.5	1.387	5.645	0.083	< 0.001
1.6	1.059	6.005	0.145	< 0.001
1.7	0.753	6.348	0.226	< 0.001
1.8	0.464	6.674	0.321	< 0.001
1.9	0.191	6.987	0.424	< 0.001
2.0	−0.068	7.288	0.527	< 0.001

Results of the Mahalanobis distance matching metric are reported in (Additional file 1: Table S4). The effect estimates are very similar to those of the propensity score matching (Table 2). Similarly, there was no qualitative difference in the results when estimating the satisfaction index using PCA (Additional file 1: Table S5).

## Discussion

This study investigated the effects of capitation on prevalence of out-of-pocket health payments and perceived service quality in insured clients using Ghana’s capitation pilot as a case study. Propensity score matching was used to balance distributions of observed covariates between NHIS insured clients who were exposed to capitation and their unexposed counterparts.

We found that exposed NHIS insured clients had higher probability of encountering out-of-pocket health payments than their unexposed counterparts. With respect to the sensitivity analysis for this estimate, DiPrete and Gangl [36] and Becker and Caliendo [37] advised that results from Rosenbaum bounds sensitivity analysis are “worst case scenarios” and that the point at which the results become sensitive to possible bias should not be interpreted as the presence of bias or the absence of a true exposure effect on the outcome. Both authors emphasised that the results from such analysis only indicate the point at which an effect estimate would become non-significant due to the influence of possible unmeasured confounders.

It is unclear whether the reported out-of-pocket payments were for services or medicines not included in the NHIS benefits package. However, given that the NHIS covers up to 95% of common clinical conditions in Ghana [39] and does not have balance billing or co-payment arrangements in its design, it is very unlikely that these reported payments were for services/medicines not covered by the NHIS. It is more likely that the out-of-pocket payments took the form of informal payments to providers. A major criticism levied against the capitation pilot from health providers was that the capitated rate was too low [4, 40]. It is therefore plausible that health facilities might have developed ways to supplement or compensate for any loss or perceived loss in revenue. Particularly, as the capitated rate was not risk adjusted, service providers with a disproportionate number of high-risk insured clients such as the elderly and those with chronic diseases would have been faced with even higher financial risk and therefore more likely to shift some of the risk to insured clients in the form of informal co-payments. Alternatively, the exposed insured clients might not have complied with the requirement under capitation to first visit their PPP when ill and might have bypassed their PPP to access healthcare from other facilities and therefore had to use alternative payments including out-of-pocket. Agyei-Baffour et al. [41] explored the perceptions and expectations of NHIS insured clients in the capitated region and reported that most insured clients cited the restriction to a single facility under capitation as a disadvantage of the payment reform.

In contrast to the out-of-pocket payments, we did not find any evidence of significant differences between the two exposure groups in any of the three perceived quality outcomes. Our findings are consistent with the findings by Andoh-Adjei et al. [14] and other studies that have investigated perceived health service quality between different groups in Ghana [27, 28, 42], but differ from those of Tangcharoensathien and colleagues [13] who found that insured clients under capitation were less likely to be satisfied with healthcare delivery compared to those under different payment methods. Given the high perceived quality ratings by both exposure groups (Table 1), our findings may suggest a possible spill over effect of the capitation pilot in the Ashanti region. The National Health Insurance Authority (NHIA, the governing body of the NHIS) made clear during the pilot its intention of a nationwide roll-out. Health service providers in the other regions might have enhanced their interpersonal relationships with NHIS insured clients with the expectation that they would register with their facilities when capitation was eventually implemented nationwide.

It is unclear if capitation was able to induce competition in health facilities in the pilot region as was anticipated by the NHIA. The ability for capitation to induce competition may depend on availability of adequate choice of health providers for insured clients. Hence, in settings where there are limited or no alternative health facilities to choose from or switch to, as is likely to be the case in most rural areas in Ghana, the conditions necessary for competition among facilities may be lacking or limited and therefore unable to induce the desired competition among health providers. In such settings, policy actors may need to consider explicitly integrating some quality benchmarks, including those of quality perceptions, into capitation designs to encourage health providers to deliver quality care.

The current study has some limitations. First, we assumed that respondents’ insurance status at the time of the survey interview was the same when they accessed the reported healthcare and that they accessed the care in their reported residing regions. These assumptions may not be true for all respondents. However, any such occurrences are likely to be randomly distributed between the two exposure groups and therefore unlikely to differentially bias the findings. Second, as implied in our definition of exposure to capitation, regional-level confounding could not be adjusted for. If there were any region-specific factors that affected the outcomes beyond the payment method difference, then those factors could introduce some bias in our estimates. Also, we could not adjust for some facility-level potential confounders such as level or size of health facility due to data limitations. For the out-of-pocket outcome however, the sensitivity analysis showed the extent of influence any unobserved or omitted variables must have on the allocation process to make the inference non-significant. Third, the study may suffer from “courtesy bias” [5]; a phenomenon where Ghanaian patients typically express satisfaction with healthcare services out of the respect they tend to have for healthcare providers and not necessarily a reflection of their true experience with the healthcare delivery. Finally, as we did not incorporate the survey sampling weights in our analyses, our findings may not be generalizable to the target population of the G-DHS. The results should therefore be interpreted within the context of these limitations.

## Conclusion

In the Ghanaian context, our findings suggest capitation was associated with a greater probability of out-of-pocket payments, and no effect on perceived service quality. Future research should examine clinical quality of healthcare and intensity of out-of-pocket payments under capitation.

## Supplementary information

**Additional file 1.** Tables S1 – S5.

**Additional file 2.** Manuscript’s dataset – data for the sub-sample analysed in this study.

## Data Availability

The full G-DHS datasets can be accessed at the DHS Program’s website (https://dhsprogram.com/Data/) after a short registration process. Data for the sub-sample analysed during this study is included within the article and its additional files.

## Abbreviations

DHS: Demographic and Health Survey
G-DHS: Ghana Demographic and Health Survey
G-DRG: Ghana Diagnostic Related Groupings
NHIA: National Health Insurance Authority
NHIS: National Health Insurance Scheme
PPP: Preferred Primary-care Provider
