# Supplementary material for: Does capitation affect patient satisfaction and prevalence of out-of-pocket payments in the insured? A propensity score analysis of Ghana’s demographic and health survey data
Source: BMC Health Serv Res. 2019 Oct 22;19:732. doi: 10.1186/s12913-019-4581-4 (PMC6805416; doi:10.1186/s12913-019-4581-4)
Supplement: Supplementary file 1 — Additional file 1. Tables S1 – S5. [file 12913_2019_4581_MOESM1_ESM.docx]

#### Table S1: Patient satisfaction scale items

| **No.** | **Item** |
| --- | --- |
| 1 | How convenient were the opening hours of the facility during the day for you? |
| 2 | How easy was it for you to see the health personnel? |
| 3 | How satisfied were you with the time you waited for your turn? |
| 4 | How satisfied were you with the time you spent in consulting or examining room? |
| 5 | How satisfied were you with the time you waited for test to be performed? |
| 6 | How satisfied were you with the time you waited for test results? |
| 7 | How satisfied were you with the time you waited at the dispensary/pharmacy? |
| 8 | How satisfied were you with the staff of the facility when they listened to you? |
| 9 | How satisfied were you with the staff of the facility when they explained what you wanted to you? |
| 10 | How satisfied were you with the staff of the facility when they gave advice and information on options for treatment? |
| 11 | How satisfied were you with the cleanliness of the facility? |
| 12 | How satisfied were you with ease of finding where to go? |
| 13 | How satisfied were you with comfort and safety while waiting? |
| 14 | How satisfied were you with privacy during consultation? |
| 15 | How satisfied were you with confidentiality and management of personal information? |

**Note**: Items are extracted and paraphrased from the 2014 G-DHS questionnaire. All items are measured on a five-point Likert scale where higher values reflect higher or better experience. The original scale in the G-DHS dataset used a reversed coding.

#### Table S2: Logit model results for the estimation of exposure probabilities

| Variable | Coefficient | S.E.^a^ | P-value |
| --- | --- | --- | --- |
| **Age group (Ref. 15-19)** |  |  |  |
| 20-24 | -0.411 | 0.324 | 0.205 |
| 25-29 | -0.418 | 0.305 | 0.170 |
| 30-34 | -0.010 | 0.307 | 0.973 |
| 35-39 | -0.401 | 0.338 | 0.235 |
| 40-44 | 0.156 | 0.337 | 0.643 |
| Over 44 | -0.206 | 0.364 | 0.572 |
| **Level of Education (Ref. None)** |  |  |  |
| Primary | 0.168 | 0.385 | 0.662 |
| Secondary/Higher | 0.389 | 0.321 | 0.226 |
| **Wealth Quintile (Ref. Poorest)** |  |  |  |
| Poorer | 0.913 | 0.434 | 0.035 |
| Middle | 1.218 | 0.429 | 0.005 |
| Richer | 2.278 | 0.436 | 0.000 |
| Richest | 2.662 | 0.472 | 0.000 |
| **Ethnicity (Ref. Akan)** |  |  |  |
| Ewe | -2.694 | 0.592 | 0.000 |
| Mole-Dagbani | -0.479 | 0.248 | 0.054 |
| Other | -0.452 | 0.224 | 0.044 |
| Resides in an urban area (Ref. rural) | -0.730 | 0.246 | 0.003 |
| Been told to have hypertension (Ref. No) | -0.092 | 0.260 | 0.722 |
| Visited a private facility (Ref. Public/Gov’t) | 0.503 | 0.189 | 0.008 |
| Constant | -3.547 | 0.492 | 0.000 |
| Number of Observations | 2,240 |  |  |
| Log likelihood | -528.656 |  |  |
| Pseudo R^2^ | 0.1426 |  |  |

**^a^** Standard error

#### Table S3: Comparisons of covariates between exposure groups before and after matching

|  |  | **Mean** | |  |  |  |
| --- | --- | --- | --- | --- | --- | --- |
| **Variable** | **Sample** | **Exposed** | **Unexposed** | **Bias (%)** | **(%) Bias Reduction** | **Variance Ratio** |
| **Age Group (Ref. 15-19)** | |  |  |  |  |  |
| 20-24 | Before Matching | 0.131 | 0.166 | -9.9 |  | 0.826 |
|  | After Matching | 0.131 | 0.131 | 0 | 100 | 1.000 |
| 25-29 | Before Matching | 0.188 | 0.202 | -3.7 |  | 0.950 |
|  | After Matching | 0.188 | 0.188 | 0 | 100 | 1.000 |
| 30-34 | Before Matching | 0.188 | 0.163 | 6.5 |  | 1.124 |
|  | After Matching | 0.188 | 0.216 | -7.5 | -15 | 0.900 |
| 35-39 | Before Matching | 0.119 | 0.145 | -7.7 |  | 0.850 |
|  | After Matching | 0.119 | 0.136 | -5 | 34.5 | 0.892 |
| 40-44 | Before Matching | 0.142 | 0.099 | 13.1 |  | 1.369 |
|  | After Matching | 0.142 | 0.119 | 7 | 46.8 | 1.160 |
| Over 44 | Before Matching | 0.108 | 0.112 | -1.4 |  | 0.970 |
|  | After Matching | 0.108 | 0.108 | 0 | 100 | 1.000 |
| **Education (Ref. None)** |  |  |  |  |  |  |
| Primary | Before Matching | 0.091 | 0.163 | -21.7 |  | 0.610 |
|  | After Matching | 0.091 | 0.102 | -3.4 | 84.2 | 0.900 |
| Secondary/Higher | Before Matching | 0.824 | 0.598 | 51.3 |  | 0.607 |
|  | After Matching | 0.824 | 0.824 | 0 | 100 | 1.000 |
| **Wealth Quintile (Ref. Poorest)** | |  |  |  |  |  |
| Poorer | Before Matching | 0.097 | 0.176 | -23.4 |  | 0.604 |
|  | After Matching | 0.097 | 0.085 | 3.3 | 85.8 | 1.119 |
| Middle | Before Matching | 0.142 | 0.208 | -17.5 |  | 0.743 |
|  | After Matching | 0.142 | 0.153 | -3 | 82.9 | 0.938 |
| Richer | Before Matching | 0.318 | 0.190 | 29.6 |  | 1.415 |
|  | After Matching | 0.318 | 0.318 | 0 | 100 | 1.000 |
| Richest | Before Matching | 0.392 | 0.163 | 52.7 |  | 1.754 |
|  | After Matching | 0.392 | 0.392 | 0 | 100 | 1.000 |
| **Ethnicity (Ref. Akan)** |  |  |  |  |  |  |
| Ewe | Before Matching | 0.017 | 0.146 | -48.4 |  | 0.135 |
|  | After Matching | 0.017 | 0.017 | 0 | 100 | 1.000 |
| Mole-Dabgani | Before Matching | 0.153 | 0.293 | -33.9 |  | 0.631 |
|  | After Matching | 0.153 | 0.148 | 1.4 | 95.9 | 1.032 |
| Other | Before Matching | 0.176 | 0.221 | -11.2 |  | 0.847 |
|  | After Matching | 0.176 | 0.148 | 7.1 | 36.6 | 1.153 |
| Residing in an urban area (Ref. Rural) | Before Matching | 0.676 | 0.508 | 34.7 |  | 0.881 |
|  | After Matching | 0.676 | 0.676 | 0 | 100 | 1.000 |
| Been told to have hypertension (Ref. No) | Before Matching | 0.142 | 0.108 | 10.3 |  | 1.271 |
|  | After Matching | 0.142 | 0.131 | 3.4 | 66.6 | 1.073 |
| Visited a private facility (Ref. Public/Gov’t) | Before Matching | 0.313 | 0.157 | 37.3 |  | 1.632 |
|  | After Matching | 0.313 | 0.290 | 5.4 | 85.4 | 1.044 |
| **Propensity score** | Before Matching | 0.154 | 0.072 | 100 |  | 1.420 |
|  | After Matching | 0.154 | 0.154 | 0 | 100 | 1.000 |

**Table S4: Effect estimates using the Mahalanobis distance matching metric**

|  | **Before Matching** | | |  | **After Matching** | | | | | |
| --- | --- | --- | --- | --- | --- | --- | --- | --- | --- | --- |
| Outcome | Crude^a^ Mean Diff. | t-stat | P-value |  | SATET^b^ | S.E.^c^ | z-stat | P-value | 95% Confidence Interval | |
| Prevalence of out-of-pocket | 0.169 | 6.15 | <0.001 |  | 0.099 | 0.041 | 2.44 | 0.015 | 0.019 | 0.178 |
| *Perceived Quality Outcomes* | |  |  |  |  |  |  |  |  |  |
| Patients' Satisfaction | 1.429 | 2.16 | 0.031 |  | 0.420 | 0.865 | 0.49 | 0.626 | -1.270 | 2.110 |
| Perceived friendliness of health staff | 0.020 | 1.13 | 0.260 |  | 0.007 | 0.016 | 0.42 | 0.676 | -0.025 | 0.039 |
| Perceived adequacy of consultation time | -0.013 | -0.61 | 0.541 |  | 0.002 | 0.027 | 0.07 | 0.941 | -0.050 | 0.054 |

^a^Unadjusted mean difference; ^b^Sample average treatment effect on the treated; ^c^Robust standard errors

**Table S5: Effect estimate on overall patient satisfaction (PCA^a^ satisfaction index)**

| Outcome | SATET^b^ | S.E.^c^ | z-stat | P-value | 95% Confidence  Interval | |
| --- | --- | --- | --- | --- | --- | --- |
| Patients’ satisfaction | 0.234 | 0.286 | 0.82 | 0.414 | -0.327 | 0.795 |

^a^Principal component analysis; ^b^Sample average treatment effect on the treated; ^c^Robust standard errors

**Note**: Propensity score matching (PSM) is used for the estimates.
